# A Cycle Slip Detection Framework for Reliable Single Frequency RTK Positioning

**DOI:** 10.3390/s20010304

**Published:** 2020-01-06

**Authors:** Salma Zainab Farooq, Dongkai Yang, Echoda Ngbede Joshua Ada

**Affiliations:** 1 School of Electronic and Information Engineering, Beihang University (BUAA), Beijing 100191, China; edkyang@buaa.edu.cn (D.Y.); ngbede@buaa.edu.cn (E.N.J.A.); 2 Department of Electrical Engineering, Institute of Space Technology, Islamabad 44000, Pakistan

**Keywords:** cycle slip detection, least squares adjustment, minimal detectable bias, RTK, reliability

## Abstract

Single frequency real-time kinematic (RTK) positioning is expected to be the leading implementation platform for a variety of emerging GNSS mass-market applications. During RTK positioning, the most common source of measurement errors is carrier-phase cycle slips (CS). The presence of CS in carrier-phase measurements is tested by a CS detection technique and correspondingly taken care of. While using CS prone measurement data, positioning reliability is an area of concern for RTK users. Reliability can be linked with the CS detection scheme through a least squares (LS) adjustment process. This paper proposes a CS detection framework for reliable RTK positioning using single-frequency GNSS receivers. The scheme uses double differenced measurements for CS detection via LS adjustment using a detection, identification, and adaptation approach. For reliable positioning, the procedure to link the detection and identification stages is described. Through tests conducted on kinematic data, internal and external reliability are theoretically determined by calculating minimal detectable bias (MDB) and marginally detectable errors, respectively. After introducing CS, the actual values of MDB are found to be four cycles, which are higher than the theoretically obtained values of one and two cycles. Although CS detection for reliable positioning is implemented for single-frequency RTK users, the proposed procedure is generic and can be used whenever CS are detected through statistical tests during LS adjustment.

## 1. Introduction

Nowadays, highly precise positioning and navigation solutions are obtained by using carrier-phase based positioning techniques such as real-time kinematic (RTK) positioning [1]. High precision is achieved once the initial phase ambiguities, inherent with carrier-phase observations, are resolved [2]. However, owing to signal blockage by obstacles and receiver motion, the continuous tracking of GNSS carrier-phase signals between two consecutive measurement epochs might get interrupted. Such a tracking loss, termed as a cycle slip, introduces a bias in carrier-phase measurements. As a consequence of cycle slips (CS), carrier-phase ambiguities need to be resolved again to avail RTK positioning accuracy. Single-epoch ambiguity resolution is very challenging in RTK because of receiver dynamics and the time-sensitive nature of the kinematic solution. Therefore, instead of resolving ambiguities again, it is beneficial if CS are detected and the corresponding measurements are taken care of [3,4]. CS detection techniques are able to perform better for dual-frequency receivers due to their ability to eliminate ionospheric effects on measurements. However, single-frequency receivers occupy more than 60% share of the current GNSS receiver market [5]. Coupled with the demand for higher positioning accuracies for commercial users, the focus has now shifted to using single-frequency receivers for providing accurate and precise navigation solutions for the mass market [6,7,8]. Single-frequency differential positioning is expected to be the primary tool for a plethora of consumer applications in areas as varied as geomatics, precision agriculture, location-based services, internet of things, and mHealth [9].

For the wide variety of RTK commercial applications, quality control of received data in real-time positioning is an area of concern [10,11]. This is due to GNSS data being subjected to measurement errors such as CS, which, if not detected, significantly degrade overall system performance [12]. Quality control involves the estimation of variances, quality tests of significance on variances, and possibly other checks [13]. In geodesy, the quality of a navigation solution can be catered to by the least squares (LS) adjustment theory. The LS adjustment theory deals with an optimal combination of unknown measurements, together with the combination of unknown parameters [14,15,16]. Using this theory, it has been established that, given the availability of redundant measurements, measurement biases can be detected by using statistical tests in connection with the adjustment of networks [17]. For positioning using code-pseudoranges, several receiver autonomous integrity monitoring (RAIM) techniques have been developed for GNSS data quality monitoring based on adjustment theory [18,19,20]. Measurement quality control is practiced by assessing, detecting, and isolating failure situations through a fault detection and exclusion procedure [18]. By applying RAIM techniques, the quality of the position fix can also be quantified during the design procedure. In this respect, the important aspects to consider are the quality of the position fix result under nominal conditions (precision) and the sensitivity of the position fix to undetected model errors (reliability) [21]. Although RAIM was traditionally designed for systems utilizing code-phase measurements for positioning, these techniques have recently been applied to CS detection in carrier-phase based positioning schemes [22]. This CS detection approach can be further extended to incorporate the evaluation of reliability in position fixing.

The concept of reliability was introduced by Baarda [14] in the context of statistical testing for outlier detection during LS adjustment for the determination of the navigation solution. As per definition, the strength of a system model depends on the level of confidence one has in the outcomes of the statistical tests. This confidence is monitored by the reliability of the fix [23]. Specifically, reliability refers to the ability of the system to detect measurement outliers (internal reliability) and the effect that undetectable outliers have on the estimated values derived from these measurements (external reliability) [23,24]. Internal reliability is defined in terms of minimal detectable bias (MDB) [23]. These are the biases that may be found with a certain probability in the outlier test. External reliability is defined in terms of the marginally detectable error (MDE) [11]. It is the influence of undetected bias on the final result of a geodetic adjustment. The statistical testing procedure presented in [14] was broken down into three parts, namely, detection, identification, and adaptation (DIA) by Teunissen [25]. It is seen that to determine the reliability of a position fix, the statistical tests in detection and identification steps are interrelated [14,23,25].

For single-frequency receivers, statistical testing during LS adjustment was used for CS detection in references [22,26,27,28,29,30,31]. All the implemented schemes were able to detect CS for an MDB of one cycle [9]. However, none of the mentioned schemes have discussed MDE, whereas it is recommended that the reliability measure of a differential position fix should be expressed in terms of external reliability [11]. Similarly, the relationship between the statistical tests during detection and identification is not considered; as a result, the reliability of the position fix cannot be asserted for any of the CS detection techniques for single-frequency receivers. This paper aims to bridge this void by presenting a detailed procedure to detect CS and determine a reliable position fix for single-frequency RTK positioning. The process is led by the DIA procedure. The chosen level of reliability is achieved by deriving decision thresholds in detection and identification stages through equating their non-centrality parameters determined from their respective probability density function (PDF). The proposed framework is tested on two kinematic datasets, and theoretical values of MDB and MDE are obtained. After CS are introduced in carrier-phase measurements, it is seen that they can be detected and a reliable position fix is obtained given the magnitude of CS is four cycles or more. It is observed that although the theoretical value of MDB is one to two cycles, the actual values are slightly higher. This is mainly attributed to the value of detection and identification thresholds determined from the chosen level of significance in the local test.

The proposed framework provides an in-depth procedure for incorporating the concept of positioning reliability with CS detection for single-frequency receivers. The flow of the paper is as follows. Section 2 discusses the CS detection algorithm for single-frequency RTK positioning. The single-frequency RTK positioning model developed for this research is introduced, followed by the LS adjustment and CS detection process through the DIA framework. In Section 3, the concept of positioning reliability is introduced, and the process of determining a reliable position fix by exploring the relationship between decision thresholds in the statistical tests for detection and identification is presented. Section 4 presents the results of the proposed framework. It describes the experimental setup, choice of parameters, and the magnitude of detected CS both theoretically and in practice. The discussion is concluded in Section 5.

## 2. CS Detection for Single-Frequency RTK Positioning

### 2.1. Single Frequency RTK Model

The code-phase and carrier-phase measurements, for a receiver r, at an epoch t, can be expressed as a pseudorange observation (in units of meters) as follows [1,2]:

(1) $$\begin{array}{l} \rho_{r}^{s}(t)=R_{r}^{s}(t)+c\left(\delta t_{r}^{}(t)-\delta t_{}^{s}(t)\right)+I_{r}^{s}(t)+T_{r}^{s}(t)+\varepsilon_{\rho }\\ \phi_{r}^{s}(t)=R_{r}^{s}(t)+c\left(\delta t_{r}^{}(t)-\delta t_{}^{s}(t)\right)-I_{r}^{s}(t)+T_{r}^{s}(t)\;+\lambda^{s}A_{r}^{s}+\varepsilon_{\phi } \end{array}$$

where $\rho_{r}^{s}(t)$ and $\phi_{r}^{s}(t)$ represent the measured code and carrier-phase observable for satellite s; $R_{r}^{s}(t)$ is the geometric distance between the receiver and the satellite; c is the speed of light; $\delta t_{r}^{}(t)-\delta t_{}^{s}(t)$ is the clock bias representing the combined offsets of the receiver clock $\delta t_{r}^{}$ and satellite clock $\delta t_{}^{s}$ with reference to system time t; $I_{r}^{s}$ and $T_{r}^{s}$ represent delays associated with signal transmission through ionosphere and troposphere, respectively; $\lambda^{s}$ is the wavelength of carrier signal and is taken as 1/L1 for single-frequency; $A_{r}^{s}=N_{r}^{s}+\delta_{r}-\delta^{s}$ is the phase ambiguity parameter and is a sum of the carrier-phase ambiguity $N_{r}^{s}$ (in cycles) and the instrumental receiver and satellite phase delays $\delta \phi_{r}-\delta \phi^{s}$ (in cycles); and $\varepsilon_{\rho }$ and $\varepsilon_{\phi }$ are the code and carrier-phase noise terms including the multipath noise. For Equation (1), it is assumed that the following corrections are taken care of by the receiver software [2]: satellite clock correction to cater for the difference between satellite vehicle (SV) time, time group delay correction, and relativistic correction.

In RTK, the double difference (DD) model is generally used for positioning. A DD measurement is obtained by differencing measurements between base b and rover r receivers and differencing the resulting values between two satellites [1]. DD is done with respect to a reference satellite, which is generally chosen as the one with the highest elevation. Considering both code-phase and carrier-phase measurements for satellites J and K, at same carrier frequencies, the DD at an epoch t, taking J as the reference satellite, is given as

(2) $$\begin{array}{l} \rho_{br}^{JK}=R_{br}^{JK}+I_{br}^{JK}+T_{br}^{JK}+\varepsilon_{\rho }\\ \phi_{br}^{JK}=R_{br}^{JK}-I_{br}^{JK}+T_{br}^{JK}+\lambda N_{br}^{JK}+\varepsilon_{\phi } \end{array}$$

The advantage of DD is that it removes common errors and biases at both ends of the baseline such that the initial carrier-phase ambiguities are integer in nature. If the baseline is short (approximately <10 km), the atmospheric effects on measurements in Equation (2) can be ignored [1,2], and Equation (2) simplifies as [2]

(3) $$\begin{array}{l} \rho_{br}^{JK}=R_{br}^{JK}+\varepsilon_{\rho }\\ \phi_{br}^{JK}=R_{br}^{JK}+\lambda N_{br}^{JK}+\varepsilon_{\phi } \end{array}$$

The method of LS will be used for RTK positioning. Considering the base to be at a known position, the observation equations are linearized about baseline $X_{br}$. For M satellites, assuming that the first satellite is chosen as reference, from Equation (3), the linearized DD code and carrier phase equations can be written as follows [32]:

(4) $$\left[\begin{array}{c} \delta \rho_{br}^{12}\\ \vdots \\ \delta \rho_{br}^{1M} \end{array}\right]=\left[\begin{array}{c} {\left(-1_{r}^{12}\right)}^{T}\\ \vdots \\ {\left(-1_{r}^{1M}\right)}^{T} \end{array}\right]\delta X_{br}\;=\left[U_{M-1}\right]\delta X_{br}\;$$

$$\left[\begin{array}{c} \delta \phi_{br}^{12}\\ \vdots \\ \delta \phi_{br}^{1M} \end{array}\right]=\left[\begin{array}{cccc} {\left(-1_{r}^{12}\right)}^{T} & \;\lambda & 0 & 0\\ \vdots & 0 & \ddots & 0\\ {\left(-1_{r}^{1M}\right)}^{T} & 0 & 0 & \;\lambda \end{array}\right]\left[\begin{array}{c} \delta X_{br}\\ \begin{array}{c} N_{br}^{12}\\ \vdots \end{array}\\ N_{br}^{1M} \end{array}\right]\;=\left[{\begin{array}{cc} U_{M-1} & \Lambda \end{array}}_{M-1}\right]\left[\begin{array}{c} \delta X_{br}\\ \begin{array}{c} N_{br}^{12}\\ \vdots \end{array}\\ N_{br}^{1M} \end{array}\right]$$

where for k=2,3…,M the terms in Equation (4) are defined as

$\delta \rho_{br}^{1k}$ = change in DD code-phase observable
$\delta \phi_{br}^{1k}$ = change in DD carrier-phase observable
$\delta X_{br}$ = size (3×1) vector for change in baseline such that
  $$\delta X_{br}=\left[\delta x_{br},\delta y_{br},\delta z_{br}\right]$$
  ${\left(-1_{r}^{1k}\right)}^{T}$ is the three-dimensional unit vector from base to rover such that [33]
  $$\left(1_{r}^{1k}\right)=\left[\begin{array}{ccc} \frac{\partial R_{r}^{1k}}{\partial x_{r}} & \frac{\partial R_{r}^{1k}}{\partial y_{r}} & \frac{\partial R_{r}^{1k}}{\partial z_{r}} \end{array}\right]\;\mathrm{and}\;\frac{\partial R_{r}^{1k}}{\partial x_{r}}=\frac{\partial }{\partial x_{r}}\left[R_{b}^{1}-R_{b}^{k}-R_{r}^{1}+R_{r}^{k}\right]$$
$U_{M-1}$ = size (M−1)×1 vector of unit vector
$\Lambda_{M-1}$ = size (M−1)×(M−1) diagonal matrix of wavelength λ

For single-epoch single-frequency RTK positioning, the linearized code and carrier–phase equations are combined to form the LS model [32].

(5) $$\left[\begin{array}{c} \delta \rho_{br}^{12}\\ \vdots \\ \delta \rho_{br}^{1M}\\ \delta \phi_{br}^{12}\\ \vdots \\ \delta \phi_{br}^{1M} \end{array}\;\right]=\left[\begin{array}{cc} U & O\\ U & \Lambda \end{array}\right]\left[\begin{array}{c} \delta X_{br}\\ \begin{array}{c} N_{br}^{12}\\ \vdots \end{array}\\ N_{br}^{1M} \end{array}\right]\;$$

For the RTK LS model in Equation (5), the size of the measurement vector (the left side of Equation (5)), is 2M−2. The redundancy of an LS model is determined by the number of measurements minus the number of unknowns. For unresolved ambiguities, the redundancy of the model is M−4. The redundancy increases to 2M−5, once ambiguities are resolved as integers.

DD observation equations are correlated. The variance-covariance (VCV) matrix for the M−1 set of DDs is given by size (M−1)×(M−1) matrix with 2 at diagonal and 1 elsewhere as follows.

(6) $$Q_{DD,M-1}=2\sigma^{2}\left[\begin{array}{cccc} 2 & 1 & \cdots & 1\\ 1 & 2 & & 1\\ \vdots & \vdots & \ddots & \vdots \\ 1 & 1 & & 2 \end{array}\right]$$

Assuming zero correlation between code and carrier observations, the measurement VCV matrix $Q_{\delta }$ for the single frequency RTK model is given by

(7) $$Q_{\delta }=\left[\begin{array}{cc} 2\sigma_{\rho }^{2}Q_{DD,M-1}^{} & 0_{M-1}\\ 0_{M-1} & 2\sigma_{\phi }^{2}Q_{DD,M-1}^{} \end{array}\right]$$

where $0_{M-1}$ is a size (M−1)×(M−1) matrix of zeros; $\sigma_{\rho }^{2}$ and $\sigma_{\phi }^{2}$ are the variances of code-phase and carrier-phase noise, respectively. These values are assumed to be a priori known and are set at 1 m^2^ and (0.01 + 1 ppm) m^2^, respectively.

### 2.2. Least Squares Adjustment

The functional model for LS adjustment is based on a linearized Gauss—Markov model of geodetic adjustment for m measurements and n unknowns. The functional model can be written as follows [17,34]:

(8) y=Ax+v

where y is the size m×1 measurement vector; A is the full rank m×n geometry matrix determined from observation equations; x is the size n×1 unknown vector with n≤m; v is the size m×1 residual or measurement noise vector assumed to be distributed with zero mean such that E(v)=0. Assuming a correct measurement model, observational residuals indicate the extent to which the measurements agree with each other. The examination of LS residuals for the detection of erroneous data is one of the most important and effective means for quality control of geodetic networks [13].

To properly weigh observations in the adjustment process, the dispersion in measurements needs to be specified [23]. Therefore, the stochastic model for y is defined with its expected value (mean) and dispersion (variance) as

(9) $$\begin{array}{l} E\left(y\right)=Ax\\ D\left(y\right)=\sigma_{0}^{2}Q_{y} \end{array}$$

where $Q_{y}$ is the size m×m VCV matrix for observations; $\sigma_{0}^{2}$ is the a priori variance of unit-weight and plays an important role in outlier detection [16,24].

Using LS, the best linear unbiased estimate of the unknown vector x is given as $\hat{x}$ [15,35]:

(10) $$\hat{x}={\left(A^{T}Q_{y}^{-1}A\right)}^{-1}A^{T}Q_{y}^{-1}y={\left(A^{T}P_{y}^{}A\right)}^{-1}A^{T}P_{y}^{}y$$

where $P_{y}=Q_{y}^{-1}$ is the cofactor or weight matrix for observations. 

After LS adjustment, the measurement residual $\hat{v}$ is given as

(11) $$\hat{v}=y-A\hat{x}$$

Using the Gauss error propagation law, the residual VCV is given as a size m×m matrix $Q_{\hat{v}\hat{v}}$ as follows:

(12) $$Q_{\hat{v}\hat{v}}=Q_{y}-A^{T}Q_{\hat{x}\hat{x}}A$$

where $Q_{\hat{x}\hat{x}}={\left(A^{T}P_{y}A\right)}^{-1}$ is the size n×n VCV matrix for $\hat{x}$.

LS adjustment is capable of estimating $\sigma_{0}^{2}$ from observations. This estimated ${\hat{\sigma }}_{0}^{2}$ is known as the a posteriori variance of unit weight.

(13) $${\hat{\sigma }}_{0}^{2}=\frac{{\hat{v}}^{T}P_{y}^{}\hat{v}}{m-n}$$

An adjustment is said to be correct if the a priori and a posteriori variances of unit weight are statistically equal [2,16].

### 2.3. Detection, Identification, and Adaptation (DIA) Approach

CS detection is conducted using DIA approach [21,23,35]. CS are treated as outliers in carrier-phase measurements, and their presence is tested based on the theory of hypothesis testing in linear models using generalized likelihood ratio tests [17]. For CS detection, the null hypothesis $H_{0}$ is assigned to the situation where the measurements are free of CS. The alternate hypothesis $H_{a}$ refers to the situation when the measurements are contaminated by CS. The generalized likelihood ratio-test helps to choose one hypothesis over the other based on the ratio of their likelihoods. For decision making, the choice of threshold T is based on system design parameters and its value is numerically determined from the PDF of measurements. In geodesy, the probability associated with the threshold, beyond which the occurrences of test-statistics are marked as outliers, which is called the level of significance α [11]. The situation where the magnitude of outliers is so small that the data containing outliers is accepted as having none is termed as a Type II error and represented by β [11]. Correspondingly, the probability of successful outlier detection is determined by the power of the test γ=1−β.

#### 2.3.1. Detection

For CS detection, the mean-shift measurement model is employed where the presence of a measurement outlier shifts the measurement mean [23,36]. In the case of a cycle slip, the bias or mean-shift is proportional to the magnitude of the slip whereas its variance remains the same. Thus, for the alternative hypothesis $H_{a}$, the measurement model can be written in the following way:

(14) y=Ax+c∇+v

where c is a known vector of the size 1×q, which takes the form ${\left(0,\ldots ,0,1,1\ldots 1\right)}^{T}$ where the presence of 1 indicates the location of CS contaminated measurements; ∇ is an unknown error vector of size q×1 with q being the dimension of outlier [23]. Subsequently, both the null and alternate hypotheses are described as follows:

(15) $$\begin{array}{l} H_{0}:y\sim N\left(Ax,\sigma_{0}^{2}Q_{y}\right)\\ H_{a}:y\sim N\left(Ax+c\nabla ,\sigma_{0}^{2}Q_{y}\right) \end{array}$$

To test whether the adjustment model is correct, an overall model test or global test (GT) is carried out to check the validity of $H_{0}$ with respect to $H_{a}$. The GT uses variance tests such that the adjustment is assumed to be correct if the a priori and a posteriori variances of unit weight are statistically equal. Assuming $\sigma_{0}^{2}$ to be equal to 1, the variance test determines the proximity of ${\hat{\sigma }}_{0}^{2}$ to unity [11,16,24,35]. The formulation of the hypothesis test for detection would thus become [16,24]

(16) $$\begin{array}{l} H_{0}:\sigma_{0}^{2}={\hat{\sigma }}_{0}^{2}\\ H_{a}:\sigma_{0}^{2}\neq {\hat{\sigma }}_{0}^{2} \end{array}$$

The test-statistic is, therefore, written as [16,35]

(17) $$T_{m-n}=\frac{{\hat{\sigma }}_{0}^{2}}{\sigma_{0}^{2}}=\frac{{\hat{v}}^{T}P_{y}\hat{v}}{m-n}$$

**Figure 1:**
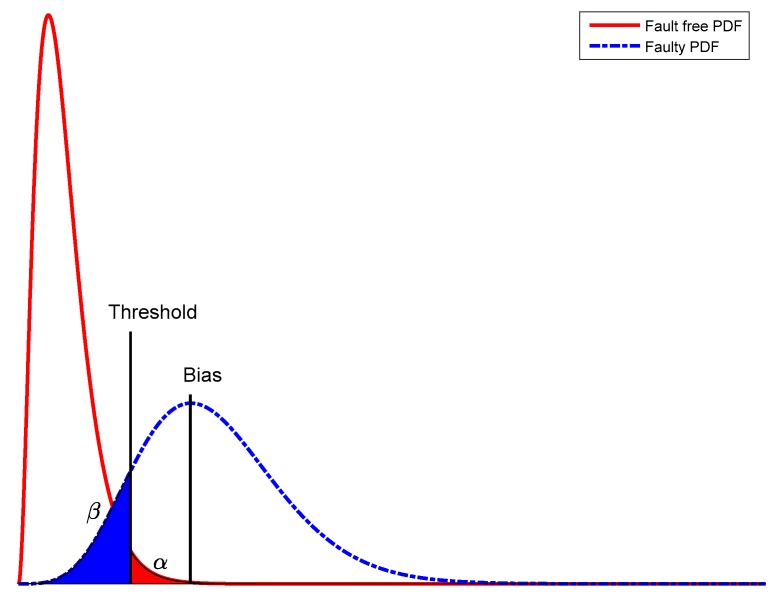
Central and non-central $\chi^{2}$ distribution for six degrees of freedom.

Thus, the test statistic is derived from the weighted sum-of-squares of LS residuals divided by the redundancy m−n. The test statistic has a Fisher or *F*-distribution distribution such that the null hypothesis will be rejected when [11,16,23,24]

(18) $$T_{m-n}>F_{\alpha }(m-n,\infty ,0)=\frac{\chi_{\alpha }^{2}\left(m-n,0\right)}{m-n}$$

where α is the chosen level of significance; F(m−n,∞,0) is the central *F*-distribution with an m−n degree of freedom for the numerator and an ∞ degree of freedom for the denominator [24]. The equality on the right side of Equation (18), $\chi_{\alpha }^{2}\left(m-n,0\right)$, is the equivalent central chi-square distribution with an m−n degree of freedom. This equality exists on the base of the relationship between *F*-distribution and $\chi^{2}$ distribution. The chi-square test is traditionally used for fault detection in RAIM. Figure 1 shows this situation for six degrees of freedom. If $H_{0}$ is rejected and $H_{a}$ is accepted, a measurement inconsistency is detected and the outlying measurement must be identified and eradicated.

#### 2.3.2. Identification

Once an error is detected, the potential source of error is identified and removed through a local test (LT). For the classical one-dimensional case (one error per adjustment), an outlier can be identified using the test statistic

(19) $$T_{q=1}=w^{2}=\frac{{\left(c_{}^{T}P_{y}{\hat{v}}_{}\right)}^{2}}{c_{}^{T}P_{y}Q_{\hat{v}\hat{v}}P_{y}c}$$

When square root is taken, Equation (19) forms Baarda’s *w*-test used in geodesy [14,23]

(20) $$w=\frac{c_{}^{T}P_{y}\hat{v}}{\sqrt{c_{}^{T}P_{y}Q_{\hat{v}\hat{v}}P_{y}c}}$$

**Figure 2:**
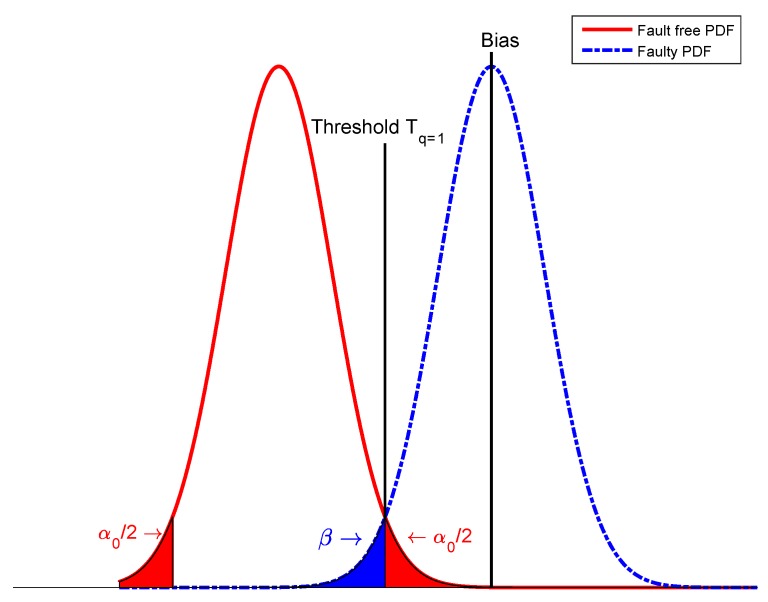
Probability density function (PDF) of the unbiased and biased normal distributions in the local test.

The *w*-test has a standard normal distribution under the null hypothesis. For a chosen level of significance $\alpha_{0}$, a model error is detected when $\left|w\right|>N_{\alpha_{0}/2}\left(0,1\right)$. This situation is shown in Figure 2.

For this framework, the usual practice of data snooping in geodesy, i.e., to check each individual observation set for potential outliers, is used. For an observation i, the test statistic $w_{i}$ reads as

(21) $$w_{i}=\frac{c_{i}^{T}P_{y}{\hat{v}}_{i}}{\sqrt{c_{i}^{T}P_{y}Q_{\hat{v}\hat{v}}P_{y}c_{i}^{}}}$$

By scanning through the whole data set, the test statistic $w_{i}$, which returns the largest value, pinpoints the observation which is most likely corrupted with a gross error. Its significance can be measured by comparing it with a critical value. The ith observation is suspected to be biased when

(22) $$\left|w_{i}\right|\geq \left|w_{j}\right|\;\mathrm{for}\;\mathrm{all}\;i\;\mathrm{and}\;\left|w_{j}\right|>N_{\alpha_{0}/2}\left(0,1\right)$$

where $\alpha_{0}$ is the level of significance of the local test. The subscript 0 indicates that it is for LT for the identification of outliers. Baarda’s *w*-test only makes a decision between the null and a single alternative hypothesis where the rejection of $H_{0}$ automatically implies the acceptance of $H_{a}$, and vice versa [37]. In general, the *w*-test is unable to detect small outliers. However, small outliers have little effect on the solution [38].

#### 2.3.3. Adaptation

Once all the sources of model error are identified, remedial action needs to be taken to get the null hypothesis accepted. The adaptation phase refers to the effective handling of the outlier such that the adjustment is satisfactory. For the proposed framework, to facilitate adaption of null hypothesis through LS adjustment, the measurement identified as an outlier is eliminated. 

## 3. Reliable Positioning

To obtain consistent high-precision positioning results with GPS carrier-phase measurements, errors unspecified in the functional or stochastic model should be correctly detected and removed or otherwise handled at the data processing stage [39]. Reliability refers to the system’s capability to detect such errors and to estimate the effects that they may have on the position. Reliability is measured by stating the size of error that might remain undetected with a specified probability [11]. Both internal and external reliability are distinguished in this respect. The internal reliability of a GNSS positioning solution is its ability to detect outliers for the chosen level of significance and power of test. External reliability informs of the impact of undetected errors on estimated positions [40]. A high internal reliability implies that small errors can be detected. High external reliability implies that statistically undetectable outliers have very little effect on the final position [11]. Reliability is driven by accuracy of observations, adjustment redundancy, and satellite geometry [16,23,35].

To ensure that the model error c∇ is reliably detected, with the same probability by both the overall model test and the *w*-test, the B-method of testing is used [14,35]. In this method, the *F*-test of the detection step and the *w*-test of the identification step are linked with each other. Given that $\lambda \left(\alpha_{m-n},m-n,\gamma_{m-n}\right)$ is the non-centrality parameter of the $T_{m-n}$ statistic for GT and $\lambda \left(\alpha_{0},1,\gamma_{0}\right)$ is the non-centrality parameter for the $T_{q=1}$ statistic for LT, the parameter $\lambda_{0}$ is given as

(23) $$\lambda_{0}=\lambda \left(\alpha ,m-n,\gamma_{m-n}=\gamma \right)=\lambda \left(\alpha_{0},1,\gamma_{1}=\gamma \right)$$

The procedure is to make a choice for $\alpha_{0}$ and $\gamma_{0}$ and calculate $\lambda_{0}$ and α from the given relationship. This choice of equal values for the non-centrality parameter $\lambda =\lambda_{0}$ and power γ=1−β in both tests implies that a certain model error can be found with the same probability by the *F*-test and the *w*-test. Both tests will, therefore, have the same reliability. Therefore, an adjustment is unreliable if after a GT failure, the LT does not fail because there is an inconsistency between the two tests, i.e., $H_{0}$ is accepted. For a chosen value of $\alpha_{0}$ for the LT, the procedure for determining values for α and the corresponding threshold in the GT is given in Table 1 [14,24,35,41]. It should be noticed that Step 1 is chosen at the design stage of the system whereas Steps 2 to 5 are conducted on an epoch by epoch basis. The values in Step 3 are derived from the monograms given in [14].

### 3.1. Internal Reliability

Internal reliability is defined as the error that can be detected by the generalized likelihood ratio test with a probability of correct detection being $\gamma_{0}=1-\beta_{0}$. It is expressed in terms of minimal detectable bias (MDB). By definition, the MDB of an alternative hypothesis is the smallest outlier that can lead to the rejection of a null hypothesis for the given probability level $\alpha_{0}$ and β [37]. Since for the proposed framework, it is assumed that only one observation is corrupted by CS at a single epoch, the following expression can be given for the MDB ∇ as [16,42]

(24) $$\left|\nabla \right|=\sqrt{\frac{\lambda_{0}\left(\alpha_{0},1,\gamma_{1}\right)}{c^{T}P_{y}Q_{\hat{v}\hat{v}}P_{y}c}}=\frac{\delta_{0}}{\sqrt{c^{T}P_{y}Q_{\hat{v}\hat{v}}P_{y}c}}$$

where $\delta_{0}$ is the shift in mean for the two hypotheses. The value for $\delta_{0}$ can be determined as [43]

(25) $$\delta_{0}=N_{1-\alpha_{0}/2}\left(0,1\right)+N_{1-\beta_{0}}\left(0,1\right)$$

It is seen that varying $\alpha_{0}$ and $\beta =\beta_{0}$ directly affects the reliability statement, so whenever an MDB is quoted, it should relate to both $\alpha_{0}$ and $\beta_{0}$ [11]. Unless the data has a very large number of outliers, any level of significance $\alpha_{0}$ from 0.1% to 5% is expected to lead to identical results [11]. On the other hand, since the MDB indicates the magnitude of outliers that can be found with a reasonable certainty, in order for the MDB to be a meaningful figure, γ has to be fairly large [11]. 

### 3.2. External Reliability

It is recommended that the reliability measure of a differential position fix should be expressed in terms of external reliability [11]. External reliability is defined as the influence of undetected bias c∇ on the final results of a geodetic computation or adjustment. It is expressed in terms of a marginally detectable error (MDE) [11]. The MDE, computed for all observations, is viewed as a measure of the capability of the network to detect blunders with probability γ [16]. A positional MDE is the effect of an undetected observational bias, with a magnitude that corresponds to the size of MDB, on the computed position [35,40,44]. The positional MDE can be determined as [40]

(26) $$\begin{array}{ll} \nabla \hat{x} & ={\left(A^{T}P_{y}A\right)}^{-1}A^{T}P_{y}c\nabla \\ & =Q_{\hat{x}\hat{x}}A^{T}c_{i}P_{y}\left(\mathrm{MDB}\right) \end{array}$$

External reliability is assessed by the largest horizontal positional MDE [11].

**Figure 3:**
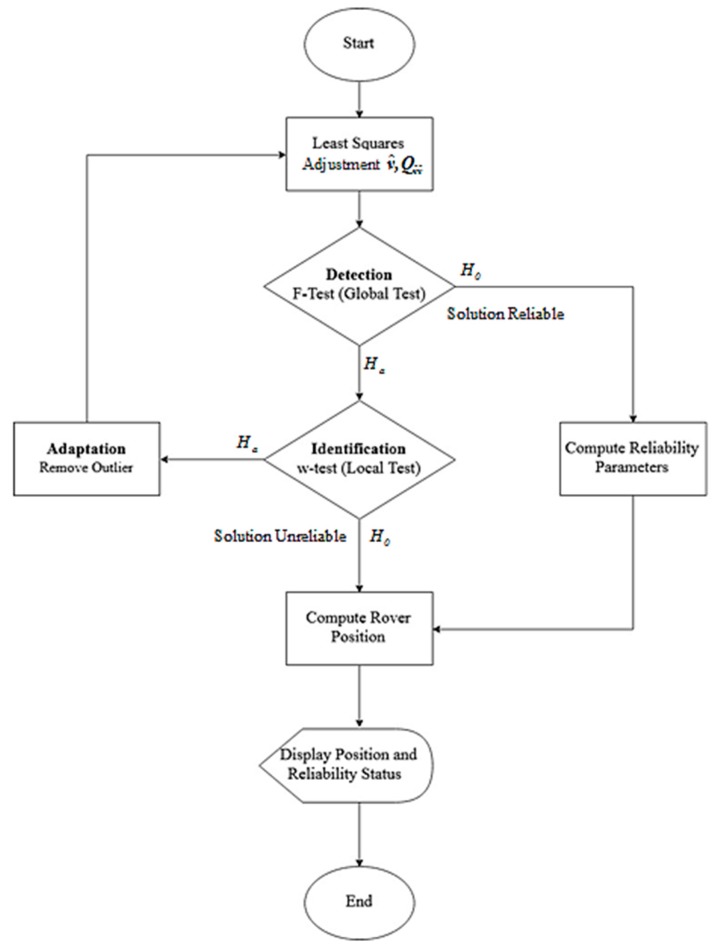
Flowchart for cycle slip (CS) detection and reliable positioning using the detection, identification, and adaptation (DIA) approach.

The framework to assess whether a position fix is reliable is represented by the flow chart in Figure 3. When there are no CS in measurements, $H_{0}$ for GT is true, the solution is deemed reliable, and the reliability parameters, i.e., the MDB for all visible satellites and the MDE values, are evaluated. For this scheme, LT is carried out for fault identification only if $H_{0}$ of the GT is rejected, and only the observation with the largest value of $w_{i}$ is tested and possibly rejected. However, once the GT fails, but no CS are identified in local test, the solution is deemed unreliable and the position fix is computed. The status as to whether the solution is reliable or not, and the case when the former is true, the MDB and MDE values, as well as the position, are displayed at the user front end.

## 4. Experimental Setup, Results, and Discussion

### 4.1. Data Collection

**Figure 4:**
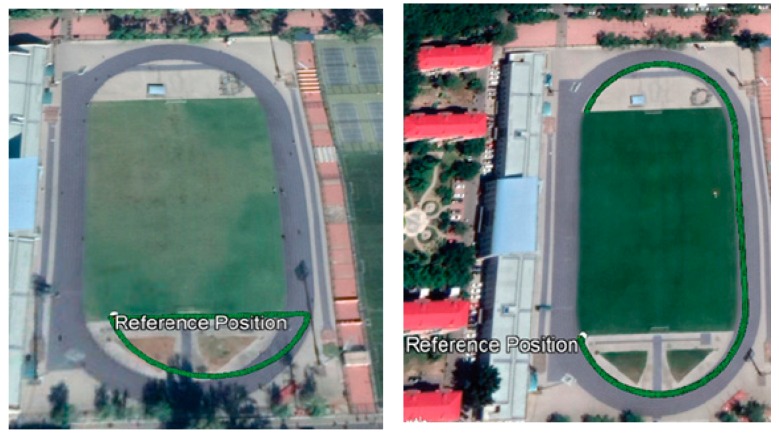
Rover receiver trajectory for dataset 1 (**left**) and dataset 2 (**right**).

For testing purposes, base and measurement data were obtained through two HUACE^®^ CHC I80 RTK receivers. Although the receiver is capable of receiving dual-frequency measurements, only L1 carrier-phase and C/A code observations are used. To reduce the multipath effect on measurements and still maintain a strong satellite geometry, an a priori elevation mask of 10° is applied for all measurements. Data were collected in the football field of Beihang University at a sampling frequency of 1 Hz for two kinematic scenarios. The details about the data are given in Table 2. For both the situations, a total of 9 GPS satellites was available. The rover path for both the scenarios is given in Figure 4 [45] where the white dot marks the reference position, i.e., the base location and the green curve plots the trajectory. Data from both receivers were acquired in HUACE propriety format. It was converted to RINEX format for post-processing. For both scenarios, base and rover data were checked to be CS-free using teqc^®^ [46]. There was no atmospheric abnormality on both days [47] and hence it was safe to assume that Equation (3) and the following single-frequency RTK model in Equation (5) could be used. For data processing, the highest elevation satellite was chosen as a reference for DD measurements.

### 4.2. Choice of Parameters 

**Figure 5:**
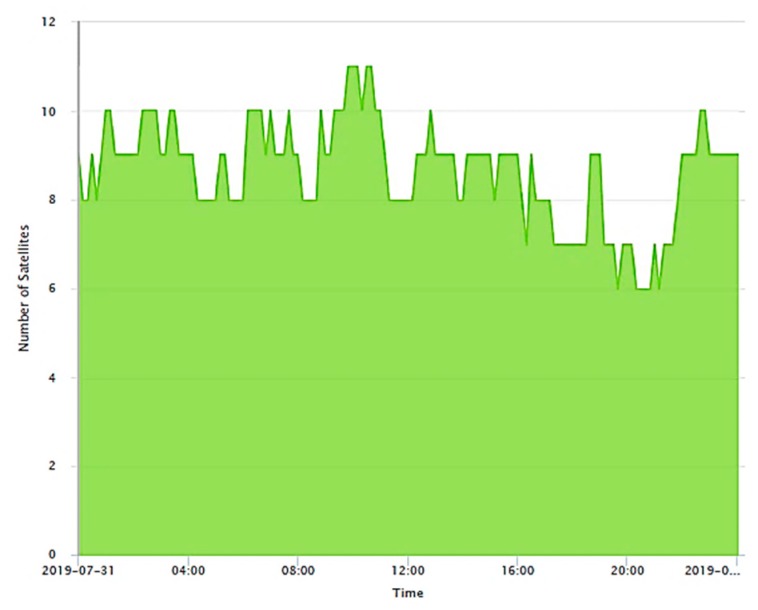
Satellite visibility plot for dataset 2.

The number of GPS satellites visible over Beijing within a 24 h duration is shown in Figure 5 for 31 July 2019 [48]. As seen, the minimum and maximum number of GPS satellites varies from 6 to 11 over the course of an entire day. CS detection takes place once ambiguities are resolved, and, corresponding to the satellite availability, the measurement redundancy varies as (7,9,11,13,15,17). From the procedure to assess the reliability of the position fix given in Table 1, the thresholds for detection and identification stages are determined. The values are listed in Table 3 for two values: $\alpha_{0}=0.1\%$ (99.9% level of confidence) and $\alpha_{0}=1\%$ (99% level of confidence). Either of these values is recommended in literature [14,16,17,35]. The recommended value of β=80% was chosen [11,23]. 

### 4.3. Results

Both datasets were evaluated for CS detection and the values of MDB and MDE were determined. A single CS was introduced midway between the datasets and was checked through the DIA procedure. The results were analysed one by one for each dataset. 

**Figure 6:**
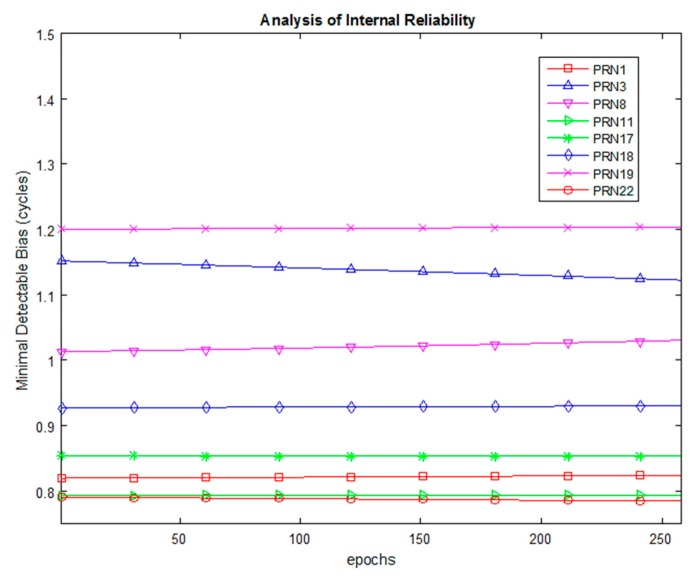
MDB for Dataset 1, $\alpha_{0}=1\%$.

**Figure 7:**
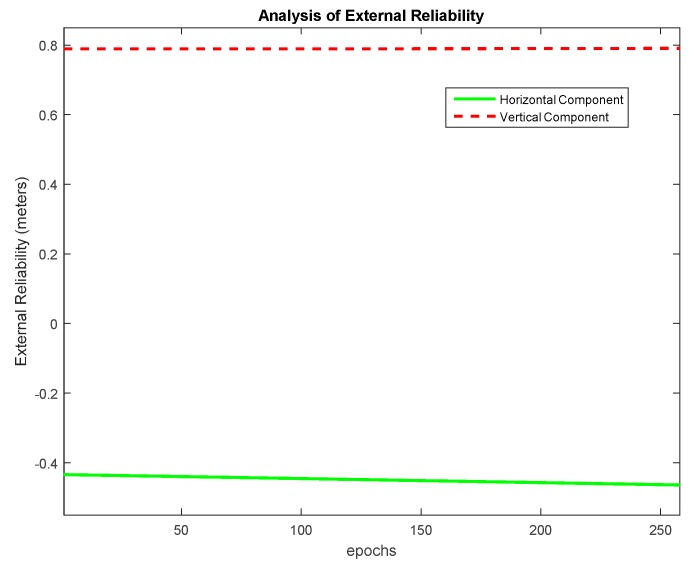
External reliability assessment for Dataset 1, $\alpha_{0}=1\%$.

The MDB values for Dataset 1 are plotted in Figure 6 for $\alpha_{0}=1\%$. The MDB values at epoch 150 and mean MDB are given in Table 4. The values are given in floating-point format as they are derived mathematically; however, the number of CS is always an integer. Hence the actual value of MDB is the ceiled number. It is seen that, theoretically, the MDB values remain between one and two cycles. It is seen that as $\alpha_{0}$ increases, the value of MDB decreases due to the decrease in magnitude of non-centrality parameter in Equation (24). This means that theoretically a smaller magnitude of CS can be detected if the level of significance is lowered. Figure 7 presents the MDE for an analysis of the external reliability for $\alpha_{0}=1\%$. Both the horizontal and vertical MDE values are plotted for illustration. As the number of satellites as well as the observable satellites remain the same during the entire course of observations, the horizontal positional MDE will remain around 0.43 m and the vertical positional MDE will remain around 0.79 m. This implies that a CS equal in magnitude to the MDB for a particular satellite would cause a positioning error equal to the MDE values quoted, i.e., for dataset 1, the occurrence of an undetected marginally detectable error in the measurements would cause a horizontal positioning error of 0.52 m in about 80% of fixes.

**Figure 8:**
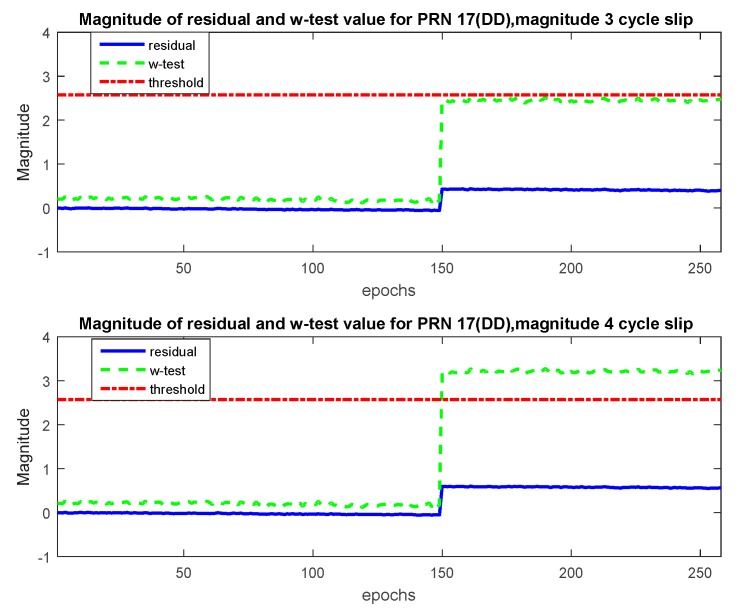
*w*-Test values for PRN 17 (dataset 1), $\alpha_{0}=1\%$.

Dataset 1 was tested for CS of Magnitude 1 to 4 at epoch 150 on PRN 17 for $\alpha_{0}=1\%$. It was observed that CS of Magnitude 1 and 2 were not detected. Figure 8 depicts the situation when CS of Magnitude 3 and 4 are introduced. As seen from the figure, the magnitude of residuals and *w*-test values are less than the threshold; however, after the introduction of CS, the values increase with the increase in *w*-test values being more profound. It can be seen from the figure that for CS of Magnitude 3, although the *w*-test values for PRN 17 were the highest, they were less than the threshold for identification. Thus the *w*-test is able to identify CS from the shift in *w*-test values. It should be noted that the CS are not removed after epoch 150; hence the offset in residuals and *w*-test values can be seen after identification. For CS of Magnitude 3, the chi-square test failed from epoch 160; however, as seen from the upper plot in Figure 8, the *w*-test values remained less than the threshold and no CS were identified. For CS of Magnitude 4, both the detection and identification stages identified the CS at the correct instant. Thus, for PRN 17, although the theoretical MDB value was two cycles, it is actually found to be four cycles for reliable RTK positioning.

**Figure 9:**
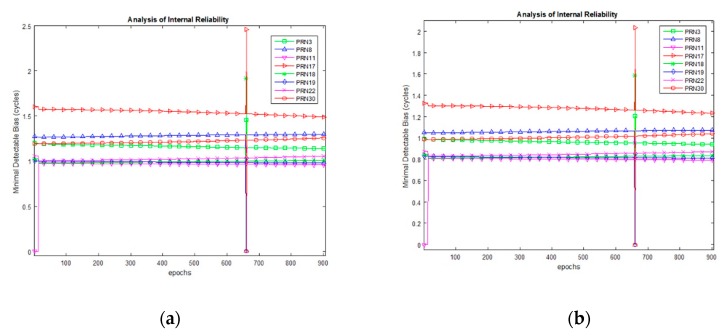
MDB for Dataset 2 for varying values of $\alpha_{0}$ (**a**) $\alpha_{0}=0.1\%$, (**b**) $\alpha_{0}=1\%$.

**Figure 10:**
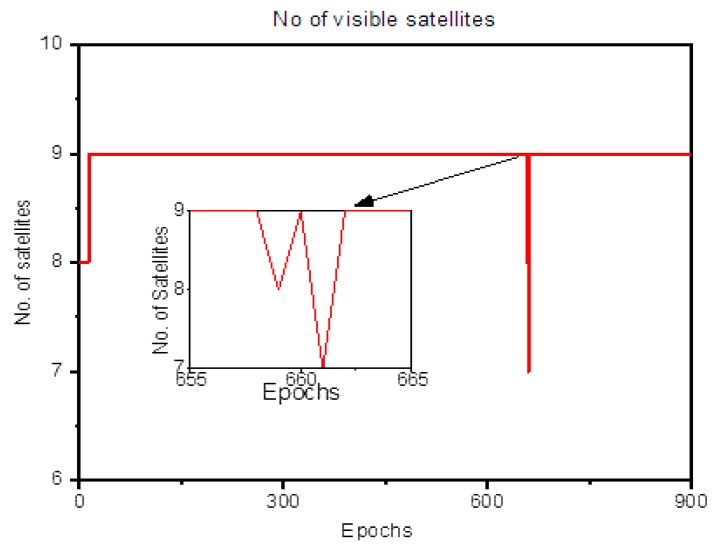
The number of visible satellites for Dataset 2.

For Dataset 2, considering $\alpha_{0}=0.1\%,1\%$, the MDB values at epoch 450 and mean MDB are given in Table 5 while the MDB values are plotted in Figure 9. PRN 11 is observed at the base at epoch 13; hence its MDB value starts from zero. As soon as the measurement joins the LS adjustment, the MDB reduces for all satellites. The number of visible satellites remains constant till epoch 658, and it changes to (8,9,7,9) for epoch (659,660,661,662); hence the peak is observed at epoch 661. This is clear from Figure 10, which shows the number of visible satellites for the rover for Dataset 2. It must be taken into consideration that the number of DD equals the number of visible satellites minus one. For the chosen value of $\alpha_{0}$, the result can be interpreted as follows:

When CS detection is carried out with a level of significance of 1% on a large dataset (906 fixes), a bias of two cycles in the DD observations for PRN 17 would be detected in about 720 (=80%) of the fixes, assuming no other sources of error are present.

**Figure 11:**
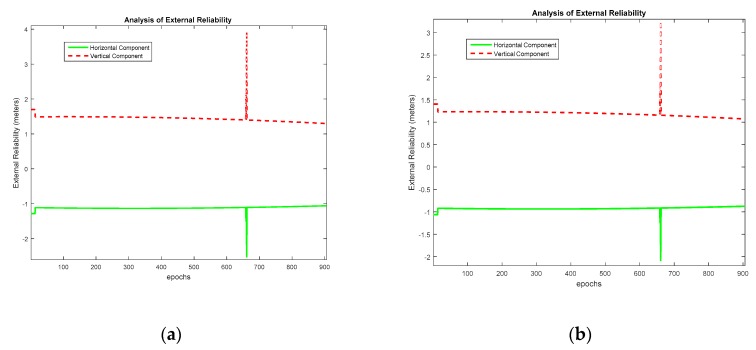
External reliability assessment for Dataset 2. (**a**) $\alpha_{0}=0.1\%$, (**b**) $\alpha_{0}=1\%$.

The MDE plot to analyze external reliability for Dataset 2 is given in Figure 11. Since MDE depends on MDB, it is seen that for the case of $\alpha_{0}=1\%$, the MDB is higher and, correspondingly, the MDE is higher than the situation when α=1%. It is seen that as long as nine satellites are visible, the horizontal positional MDE remains around 1.12 and 0.92 m for the cases when α=0.1% and α=1%, respectively.

**Figure 12:**
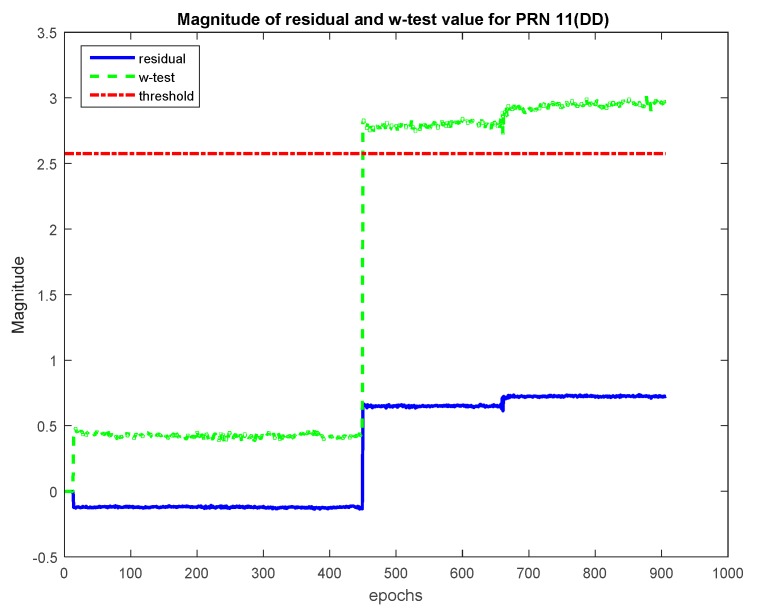
*w*-Test values for PRN 3 (Dataset 2).

For Dataset 2, at epoch 450, CS varying in magnitude from 1 to 4 cycles were introduced for $\alpha_{0}=1\%$. All the tracked satellites were checked for the minimum size of CS that could be reliably detected. Similar to Dataset 1, it was found that although the theoretical MDB varies between one and two cycles, in practice the CS of this magnitude could not be detected. Although for some satellites, CS of Magnitude 3 could be detected, but it was at a later epoch. CS of size 4 could be detected and identified for all PRNs. Table 6 lists the residual and *w*-test values for PRN 3 and PRN 11 at the epoch before and after CS of Magnitude 4 cycles. It is observed that after CS occur, the *w*-test values increased for all DD values. This is due to aggregately processing all signals through the LS adjustment process. CS contaminated measurements are identified by the largest *w*-test value. The shaded values for corresponding satellites after the occurrence of CS indeed show that their *w*-test value is largest and hence CS can be identified. In addition, it is observed that the residual for the CS contaminated satellites is also the highest. Figure 12 depicts this situation for PRN 3.

## 5. Conclusions

A framework for CS detection and determination of a reliable position fix for single-frequency RTK receivers is presented in this paper. The scheme uses DD measurements to detect CS during an LS adjustment. Once detected, CS-contaminated measurements can be eliminated from the adjustment model and position fix, along with reliability parameters MDB and MDE being computed. From the reliability assessment of the proposed scheme on two dynamic datasets, it is seen that MDB depends on the level of significance $\alpha_{0}$ chosen in the LT and the number of observed satellites. MDB increases as $\alpha_{0}$ decreases. However, the choice of $\alpha_{0}=0.1\%$ and $\alpha_{0}=1\%$ does not affect the MDB significantly. MDB increases as the number of visible satellites decreases. In addition, although theoretically the MDB is one or two cycles for the chosen values of $\alpha_{0}$ and β, in practice it is four cycles for the two scenarios. This can be attributed to measurement noise which was ignored while developing the single-frequency RTK model in Equation (5). MDB can be decreased and detection can be improved by lowering the value of $\alpha_{0}$ in the LT, which lowers decision thresholds for both the tests. However, it was seen that this causes false flags and several measurements were incorrectly identified as CS. Therefore the recommended values of $\alpha_{0}=0.1\%$ and $\alpha_{0}=1\%$ were retained. Also, it is less likely to have very small cycle slips (e.g., one to two cycles) in the data and it is usually hidden in the higher noise levels in kinematic navigation with low-cost equipment [49].

## Figures and Tables

**Table 1:** Determining the threshold for the global test.

S.No.	Step	Parameter	Procedure
1	Choose	$\alpha_{0},\gamma$	Done once at the design stage
2	Determine	Redundancy 2M−5	Calculated from number of visible satellites
3	Determine	$\lambda_{0}$	Equation (23)
4	Find	α	Monogram [14]
5	Determine	$T_{m-n}$	Equation (18)

**Table 2:** Details of datasets used for testing.

Dataset	Date (DD-MM-YY)	Day of Year	Number of Epochs	Baseline Length (Meters)	Visible Satellites (PRN)	Reference Satellite (PRN)
1	27-07-2019	208	258	3 to 66	1,3,8,11,17,18,19,22,28	28
2	31-07-2019	212	906	0.5 to 140	1,3,8,11,17,18,22,28,30	1

**Table 3:** Variation of the global test parameter α against $\alpha_{0}$ and the redundancy using the chi-square test.

Redundancy	$\alpha_{0}=0.1\%,T_{q=1}=2.58,\lambda_{0}=17.07$		$\alpha_{0}=1\%,T_{q=1}=3.29,\lambda_{0}=11.67$	
	α	$T_{m-n}$	α	$T_{m-n}$
7	0.02	4.765	0.1	7.041
9	0.035	5.411	0.125	7.493
11	0.05	5.892	0.15	7.901
13	0.06	6.163	0.175	8.278
15	0.07	6.409	0.2	8.634
17	0.08	6.634	0.25	9.299

**Table 4:** Minimal detectable biases (MDBs) for observable satellites in Dataset 2.

SV	$\alpha_{0}=0.1\%$		$\alpha_{0}=1\%$	
	MDB Epoch 150 (Cycles)	Mean MDB (Cycles)	MDB Epoch 150 (Cycles)	Mean MDB (Cycles)
1	0.994	0.994	0.822	0.822
3	1.373	1.376	1.136	1.138
8	1.236	1.234	1.022	1.021
11	0.959	0.959	0.793	0.793
17	1.032	1.032	0.854	0.855
18	1.123	1.123	0.929	0.929
19	1.454	1.453	1.202	1.202
22	0.952	0.952	0.787	0.788

**Table 5:** MDBs for observable satellites in Dataset 2.

SV	α=0.1%			α=1%		
	MDB Epoch 13 (Cycles)	MDB Epoch 450 (Cycles)	Mean MDB (Cycles)	MDB Epoch 13 (Cycles)	MDB Epoch 450 (Cycles)	Mean MDB (Cycles)
3	1.1973	1.164	1.163	0.9902	0.962	0.963
8	1.2701	1.280	1.284	1.0504	1.062	1.059
11	0	0.954	0.968	0	0.801	0.789
17	1.6028	1.544	1.549	1.3256	1.281	1.277
18	1.0235	0.996	0.993	0.8465	0.821	0.823
22	1.0024	0.988	0.988	0.8290	0.817	0.817
28	1.0053	1.026	1.022	0.8728	0.846	0.848
30	1.1922	1.216	1.231	0.9860	1.004	1.006

**Table 6:** Residuals and *w*-test values for CS introduced at epoch 450 for Dataset 2, $\alpha_{0}=1\%$.

SV	Epoch	CS Introduced in PRN 3		CS Introduced in PRN 11	
		Residual	*w*-Test	Residual	*w*-Test
PRN 3	449	−0.0343	0.1524	−0.0343	0.1524
	450	0.3548	2.8005	0.2150	0.2617
PRN 8	449	0.0671	0.0872	−0.067	0.0872
	450	0.1631	0.9327	0.1348	0.3346
PRN 11	449	−0.1334	0.4538	−0.1334	0.4538
	450	−0.0944	0.3007	0.6576	2.8105
PRN 17	449	−0.0340	0.2049	−0.0340	0.2049
	450	0.0428	0.8047	0.2563	0.7351
PRN 18	449	−0.0623	0.0383	−0.0623	0.0385
	450	−0.0214	0.1300	0.0171	0.9653
PRN 22	449	−0.0407	0.0910	−0.0407	0.0910
	450	−0.2876	1.4620	0.1296	0.2869
PRN 28	449	−0.0377	0.1127	−0.0377	0.1127
	450	0.0748	0.7290	0.0195	0.9795
PRN 30	449	−0.0937	0.2775	−0.0937	0.2775
	450	−0.2933	1.8384	0.1703	0.0549
